# Evaluation of the appearance of osteochondrosis lesions by two radiographic examinations in sport horses aged from 12 to 36 months

**DOI:** 10.1371/journal.pone.0286213

**Published:** 2023-05-23

**Authors:** Raphaël Van Cauter, Didier Serteyn, Jean-Philippe Lejeune, Alycia Rousset, Isabelle Caudron

**Affiliations:** 1 Centre Européen du Cheval, Mont-le-Soie, Vielsalm, Belgium; 2 Département des Sciences Cliniques des Équidés, Chirurgie et Orthopédie, FARAH, Université de Liège, Liège, Belgium; University of Life Sciences in Lublin, POLAND

## Abstract

Osteochondrosis is a developmental orthopedic disease characterized by a defect of enchondral ossification. This pathological condition develops and evolves during growth and is influenced by various factors, in particular genetic and environmental. However, little research has been conducted on the dynamic of this condition in horses after the age of 12 months. The retrospective study presented here investigates changes in osteochondrosis lesions through two standardized radiographic examinations carried out on young Walloon sport horses after one year of age (mean age at first and second examination was 407 (±41) and 680 (±117) days respectively). Each examination, analyzed independently by three veterinarians, included latero-medial views of the fetlocks, hocks, stifles, plantarolateral-dorsomedial hocks view and additional radiograph if the operator deemed it necessary. Each joint site was graded as healthy, osteochondrosis (OC) or osteochondrosis dissecans (OCD) affected. A group of 58 horses was studied, among them 20 presented one or more osteochondrosis lesions for a total of 36 lesions present during at least one examination. In this population, 4 animals (6.9%) presented osteochondrosis during only one examination (2 at the first examination and 2 at the second one). Moreover, it was possible to demonstrate the appearance, disappearance and more generally the evolution of 9/36 lesions (25%) within the different joints. The results of the study suggest that, although substantial main limitations, osteochondrosis lesions can evolve after the age of 12 months in sport horses. Knowing this is useful in helping to decide the appropriate radiographic diagnosis timing and management.

## Introduction

Developmental Orthopedic Diseases in equines include a series of clinical entities developed during growth with a diverse pathogenesis and variable clinical expressions. Among these is osteochondrosis (OC) [1]. This pathological condition develops during growth and is caused by a defect in articular cartilage differentiation and maturation leading to osteochondral structural abnormalities and/or fragments within the joints [2]. When a fragment is free within the joint, the lesion is referred to as “dissecans” (OCD). In this study, the abbreviation “OC(D)” is used to describe osteochondrosis lesions, no matter whether a fragment is associated or not.

Lameness is one of the leading causes of retirement in the sport and race horse industries [3, 4]. OC(D), although often non-clinical, nevertheless appears to be a significant cause of lameness [5, 6]. This pathology, described in humans and in at least 6 animal species, has therefore been the subject of considerable research [7–11]. However, its etiology remains poorly understood. It consists of a focal disturbance of the enchondral ossification process within growth cartilage. Growth cartilage is present in metaphysis where it is called “metaphyseal growth plate” in young animals but also at the end of immature joints epiphysis–it is then called “epiphyseal growth cartilage” [12]. Nowadays, different theories on the pathogenic process leading to lesions remain regarding the causes and mechanisms underlying OC(D) development. Research highlights the key role of a vascularization defect in the early development of OC(D) lesions occurring during the passage of blood vessels within the ossification front and resulting in areas of ischemic necrosis of chondrocytes [2, 12, 13]. Furthermore, histopathological observations of the epiphyseal cartilage, particularly in foals, have shown that cartilage thickness, the number of vessels and their direction follow a heterogeneous distribution which could explain, in part, the susceptibility of specific sites within the joint to develop OC(D) [14–16]. However, processes leading to a vascular failure remain poorly elucidated and various hypotheses persist -specifically an implication of the forces (compression, shearing) exerted at the level of the osteochondral junction of the ossification front [1, 12], extracellular matrix alteration [17, 18], involvement of energetic metabolism [19, 20], alteration of the chondrocyte proteome [21, 22], hormonal dysregulation [23, 24] and, in foals which suffered from bacterial infection during the first months of their lives, a process of septic ischemic chondronecrosis (9). Although the understanding of pathogenic processes leading to OC(D) has been the subject of much research, the dynamic aspect remain poorly studied. Various authors have demonstrated a regression of OC(D) lesions involving proliferation of chondrocytes and blood vessels adjacent to the injured area. However, the underlying mechanisms remain poorly understood [2, 16].

OC(D) prevalence in equines varies according to breed and studies. It seems to be around 23% in Thoroughbreds, 49% in Lusitano and around 40% in Warmblood horses [24–27]. This pathological condition can manifest in different joints. In sport horses, it is usually diagnosed in fetlocks, hocks and stifles. However, it is not exceptional to find them in other joints such carpus, cervical spines, shoulders and hips [5, 28–31]. There is currently no consensus on the lesion’s evolution period and the age of “no return”. Indeed, studies on a large number of horses report that these lesions evolve in different breeds (Thoroughbred, Standardbred, Selle-Français, Polish and Belgian Warmblood) in foals aged between 6 and 18 months and that this phenomenon is influenced by environmental and dietary factors [24, 30, 32, 33]. However, research on the precise evolution of lesions within different joints is scarce, the number of included animals and lesions is relatively low and the radiographic protocols are different [34–36]. The age at which lesions become stable differed according to the studies—however, none demonstrated a regression of lesions after 12 months of age in Warmblood horses.

The aim of this study was to determine the presence and evolution of OC(D) lesions in the metacarpo- and metatarsophalangeal, tarsocrural and femoropatellar joints in sport horses radiographed twice between 12 and 36 months of age.

## Materials and methods

### Population

A group of 58 horses (23 females and 35 males) was enrolled for this study. The distribution of horses within the stud-books was as follows: 39 SBS (Belgian Sport Horse), 5 BWP (Belgian Warmblood Paard), 5 Zangersheide, 4 AES (Anglo European Stud-Book), 2 Selle-Français, 2 Hanoverians and 1 KWPN (Koninklijk Warmbloed Paard Nederland). The horses were presented on the basis of a call for applications within the framework of a screening program for orthopedic developmental diseases supported by the Walloon Region (Belgium) to promote and help horse breeding [37]. This program was aimed at sport horses breeders in the Walloon Region and aimed at carrying out X-ray examinations on foals from birth to 36 months old. These examinations were carried out at “Centre Européen du Cheval” in Mont-le-Soie or within the breeding farm. All of the foals from a same farm were systematically examined, within the limits of their cooperation (and risks associated with their behavior). The animals were identified during the first examination with a unique number for an objective processing of the data. The breeding conditions of the population studied here were not recorded due to the related lack of statistical power associated with the low number of individuals (58) and the absence of this information in the anamnesis of the horses studied here. Moreover, breeding management of 204 foals within this program and the impact on osteochondrosis was already described in a previous study [24].

### Radiographic examinations

For each animal, two radiographic examinations were available, each exam was identified by a number (1 or 2). The first one was carried out after the foal was 12 months old (average 407 ± 41 days), and the second one before 36 months of age (average 680 ± 117 days). When first examined, the youngest individual was 365 days old and the oldest 515 days old. On second examination, the age varied between 527 days and 844 days. The number of days between examinations ranged from 142 to 405 days (average 273 ± 76 days).

Radiographic examinations included the following views: latero-medial of the 4 fetlocks, latero-medial and plantarolateral-dorsomedial oblique of the hocks and latero-medial of the stifles. Additional views were executed when the operator deemed it necessary. In order to optimize radiographic examinations, the animals were sedated with an intravenous injection of romifidine hydrochloride (0.04 mg/kg IV) together with butorphanol (0.02 mg/kg IV). X-ray sources were generated with a Gierth RHF 200 ML portable device. From 2008 to 2013, the development of fluorescent screen cassettes was carried out by a Vetray CR 2430 scanner and the examination of digital radiographs was performed with the processing Vetray Vision analysis software 4.4.4 (Vetray Gmbh, Pfaffenhofen, Germany). Between 2014 and 2020, radiographs were developed with a Examion CR Vita 45 Scanner and examined in a digital format with the Vita CR System Software V.3.2 (Carestream Health Rochester, NY, USA). From 2021 to 2022, X-rays were operated with the FUJIFILM Console Advance Software and sent to VSOL program for storage and reading (FUJIFILM Europe GmbH, Düsseldorf, Germany).

### Radiographic interpretation

Three veterinarians (a PhD candidate (Vet 1) and two veterinarians with a long experience in locomotor pathology study (Vets 2 and 3)) reviewed all radiographs. The analysis was done individually and without knowledge of what could have been observed by the other two vets. It was considered that OC lesion was present when an alteration of the contour (flattening, depression, irregularity) of the articular margin or the underlying zone (altered opacity) was demonstrated. When a fragment was present (possibly with the presence of modifications mentioned above), the lesion was classified as OCD. In case of an oval zone showing delimited loss of density within the bone, the lesion was described as cystic and included with OC lesions for prevalence. In the event of a disagreement between observers, the less severe grade (healthy<OC<OCD) was retained.

Regarding the radiographic protocol, the following sites were analyzed for OC(D) lesions:

- Metacarpophalangeal and metatarsophalangeal joints: dorso-proximal (DP) aspect of the first phalanx (P1), intermediate ridge of the condyles (IR) of the metacarpal III (MCIII) and metatarsal III (MTIII).
- Tarsocrural joint: distal aspect of the intermediate ridge of the tibial cochlea (DIRT) and Lateral trochlear ridge of the talus.
- Femoropatellar joint: lateral trochlear ridge of the femoral trochlea (LTF), Medial trochlear ridge of the femoral trochlea and condyles of the femoral trochlea.

Within metacarpophalangeal and metatarsophalangeal joints, Palmar/Plantar Osteochondral fragments (POFs) were not taken into account in the calculations. Variations in the appearance of the distal end of the medial trochlear ridge of the talus (“dew drop syndrom”) have been considered as anatomical variations and not as lesions [38].

For the evolution classification, each articulation and lesion was categorized as follows: (1) disappearance when a lesion was present only at first examination, (2) improvement when an OCD lesion at first examination was classified as OC at the second one, (3) stable when the same grade was retained at both examinations, (4) deterioration when an OC lesion at first examination was graded as OCD at the second one, (5) appearance when the lesion was not detected during the first radiographic examination.

### Statistical analysis

Statistical analyses were performed using Microsoft Excel software and XLSTAT (Assinsoft, Paris, France). Each animal was classified by a binary scale where 0 represented the absence of evolution in all joints and 1 the presence of an evolution in at least one joint. Statistical analysis of the presence of an evolution of lesions in relation to the sex, age and number of days at and between examinations were performed. The Shapiro-Wilk test was used to determine the normality of the distribution and the χ2 /Fisher’s exact test was used to compare categorical variable. The normal and non-normal distributed quantitative variables were respectively assessed by independent T-tests or Mann-Whitney tests [39]. P-value of <0.05 noted statistical significance. One sample Z-test with a significance level of 0.05 was performed to assess whether the appearance or disappearance of OC(D) within joints and individuals differed by 1% and a two sample Z-test was used if presence of OC(D) evolution was influenced by gender [40]. To ensure agreement between all 3 reviewers, interobserver agreement in diagnosing OC(D) and its evolutionary aspect was determined for each joint of the last 25 radiographed animals (200 joints) using the Kappa statistic [41].

## Results

Among the 58 horses selected in this study, 20 (34.5%) presented OC(D) at examination 1 and/or 2. In this group, 4 (6.9%) were affected during one examination only (2 at examination 1 and 2 at examination 2) which is, according to Z-test analysis, statistically different from 1%.

Among the 464 joints analyzed, 36 osteochondrosis lesions (7.8% of the joints) were observed at least during one exam (32 during the first exam and 34 during the second one) (Table 1). The proportion of lesions which disappeared (2/32) was statistically different from 1% and, on the contrary, the proportion of lesion which appeared at examination 2 (4/432) was not statistically different from 1% (analyze performed with a Z-test). Considering the evolutionary aspect of lesions present during both examinations, 1/34 (2.9%) showed an improvement of the lesion on second examination while 2/34 (5.9%) presented a deterioration of the radiographic image. Concerning OC(D) lesions detection and grading, there was a substantial agreement (κ = 0.8) between Vet 1 and Vet 2, an almost perfect agreement (κ = 0.84) between Vet 1 and Vet 3 and a substantial agreement (κ = 0.8) between Vet 2 and Vet 3. Regarding the evolutionary appearance of joints, there was a moderate agreement (κ = 0.50) between Vet 1 and Vet 2, a fair agreement between Vet 1 and Vet 3 (κ = 0.40) and a substantial agreement (κ = 0.71) between Vet 2 and Vet 3. The Statistical analysis of the presence of an evolution of the lesions in relation to the sex, age at second examination and number of days between examinations did not make it possible to highlight a significant difference. Then, the only variable which showed a significant effect (p = 0.031) was the age at first examination (Table 2).

**Table 1:** Evolution of osteochondral lesions during two radiographic examinations on 58 horses (aged 12 to 36 months).

		OC(D)			
Exam 1		+	+	-	-
Exam 2		+	-	+	-
MCP	P1	0	0	1	115
	MCIII	4	1	2	109
MTP	P1	3	0	0	113
	MTIII	10	0	1	105
TC	DIRT	6	0	0	110
FP	LL	5	1	0	110
	Cyst	2	0	0	114
Total		30	2	4	776

OC(D) = Osteochondrosis and osteochondrosis dissecans, MCP = Metacarpophalangeal joint, MTP = Metatarsophalangeal joint, TC = Tarsocrural joint, FP = Femoropatellar joint, P1 = First Phalanx, MCIII = Metacarpal III, MTIII = Metatarsal III, DIRT = Distal Intermediate Ridge of the Tibial cochlea, LR = Femoral Lateral Ridge.

**Table 2:** Statistical analysis of age at examinations 1 and 2, number of days between examinations and sex influence regarding presence or absence of OC(D) evolution.

Parameter	Statistical test	p-value calculated	Conclusion^a^
**Exam 1 age (days)**	Mann-Withney	**0,031**	H1 (age difference between evolutionary and stable OC(D) group) must be considered
**Exam 2 age (days)**	T-test	0,147	H0 (age equality between evolutionary and stable OC(D) group) cannot be rejected
**number of days between exam 1 and exam 2**	Mann-Withney	0,354	H0 (number of days equality between evolutionary and stable OC(D) group) cannot be rejected
**Sex**	Z-test	0,787	H0 (proportion equality between evolutionary and stable OC(D) group) cannot be rejected

^a^The level of significance was set at p<0.05

### Metacarpophalangeal joint

A total of 116 joints were analyzed.

**Fig 1:**
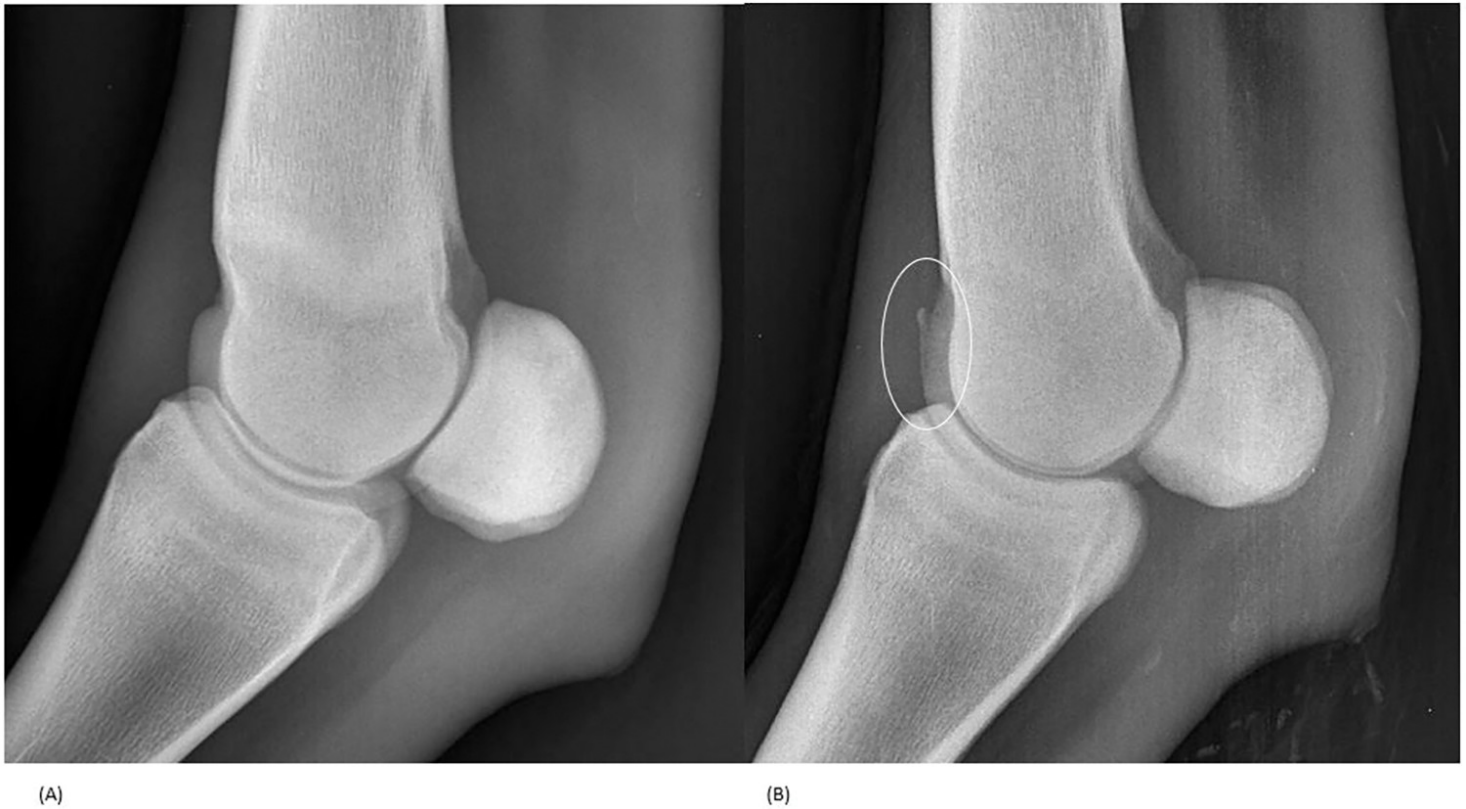
Left metacarpophalangeal latero-medial view at 433 days (A) and 715 days (B) presenting an osteochondrosis lesion appearance: (A) The joint was graded as healthy, without any signs of osteochondrosis. (B) The dorso-proximal aspect of the intermediate ridge of the metacarpal III condyles presents a radiolucent line (circled) indicating the presence of an OC lesion.

A group of 5 joints (4.3%) showed OC(D) lesions on first examination and 7 (6%) on second examination (Table 1). During both examinations, 4 OC(D) were detected, in addition, 1 was only observed on examination 1 whereas 3 were objectified only on examination 2 (Fig 1).

### Metatarsophalangeal joint

Among the 116 joints analyzed, 13 (11.2%) presented OC(D) lesions on first examination and 14 (12%) on the second one. Indeed, 1 OC of the IR of the MTIII lesion was only present at examination 2 (Table 1).

Concerning the evolutionary aspect of the lesions present on both examinations, 1 was evaluated as deteriorated.

6 joints presented a POF during both examinations (not counted in prevalence and evolution calculations). No change in their radiographic appearance could be demonstrated.

### Tarsocrural joint

**Fig 2:**
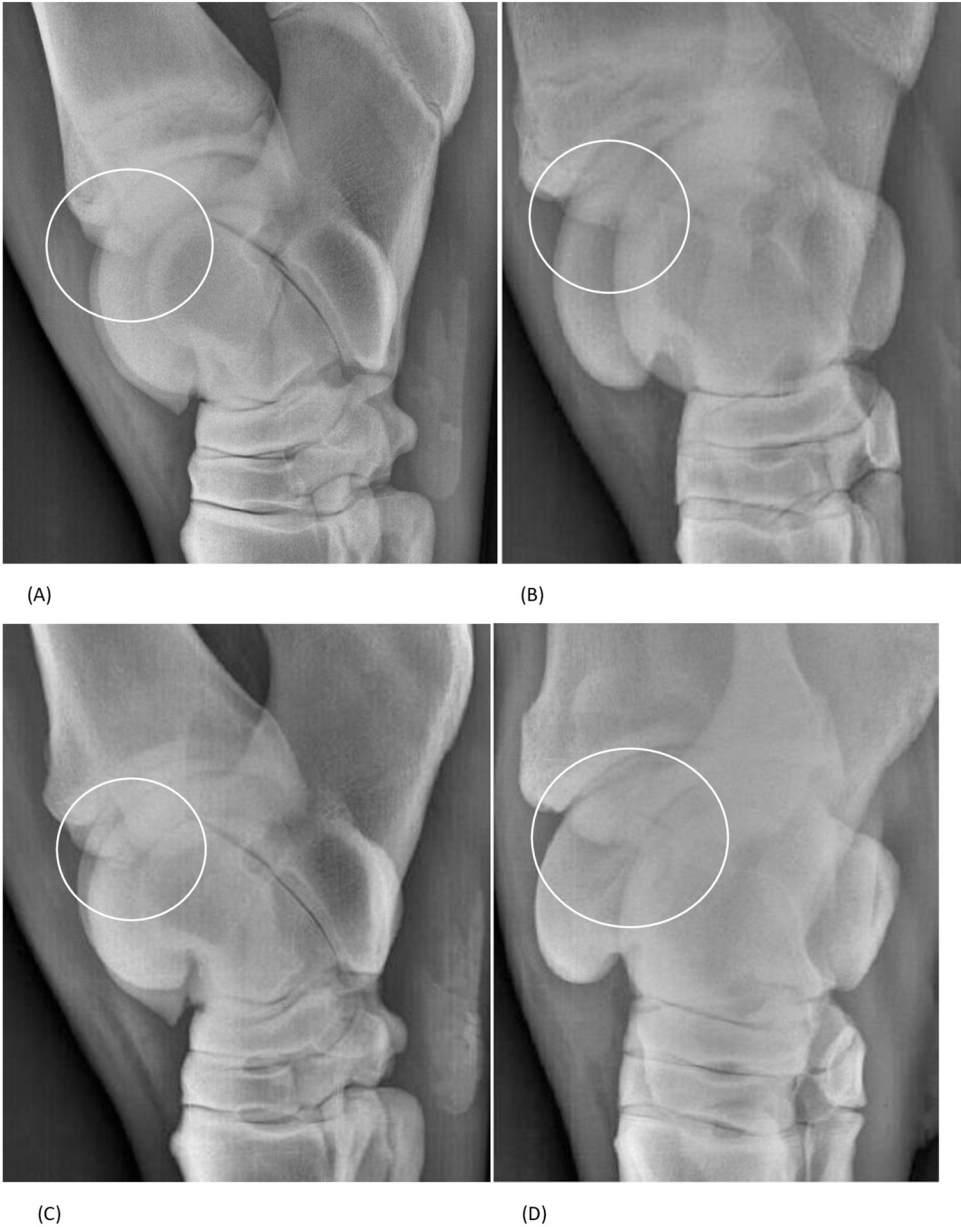
Latero-medial and plantarolateral-dorsomedial oblique view of a right tarsocrural joint at 368 days (A and B) and 726 days (C and D): (A) and (B) The distal end of the intermediate ridge of the tibial cochlea shows decreased density (circled) indicating the presence of osteochondrosis. (C) and (D) Presence of a notch and an associated osteochondral fragment (circled) in the distal intermediate ridge of the tibial cochlea. The lesion was considered to have deteriorated (classified as osteochondrosis dissecans).

Out of the 116 joints evaluated, only lesions of the DIRT were identified. 6 (5.2%) lesions were present during both examinations: 5 OCD and 1 OC on first examination and 6 OCD on the second one (Table 1). Thus, an OC lesion on first examination evolved into OCD (Fig 2).

In terms of the evolution of the radiographic appearance, only the OC lesion then taking on an OCD appearance was considered to be deteriorated.

### Femoropatellar joint

Among the 116 joints available, 8 (6.9%) OC(D) lesions were identified on first examination and 7 (6%) on the second one (Table 1).

**Fig 3:**
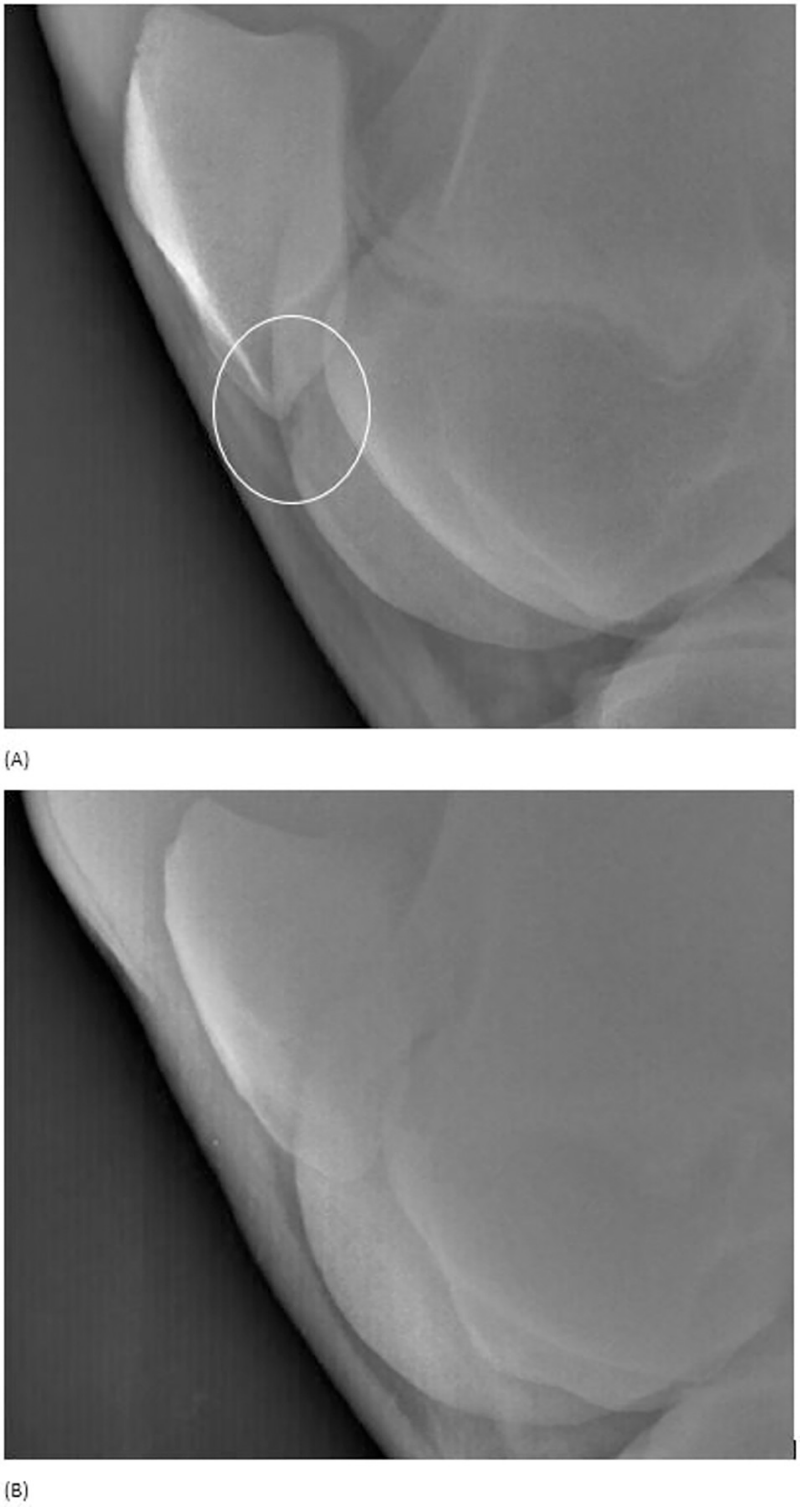
Caudolateral-craniomedial oblique view of the right femoropatellar joint of a horse at 376 days (A) and 710 days (B): (A) Loss of density within the lateral ridge of the femoral trochlea in the subpatellar region (circled) suggesting an osteochondrosis lesion. (B) The density of the lateral ridge is completely homogeneous. The lesion was considered to have disappeared.

Among the 4 LTF OC on first examination, 3 were still present on second examination and one lesion showed complete resolution of its radiographic appearance (Fig 3).

**Fig 4:**
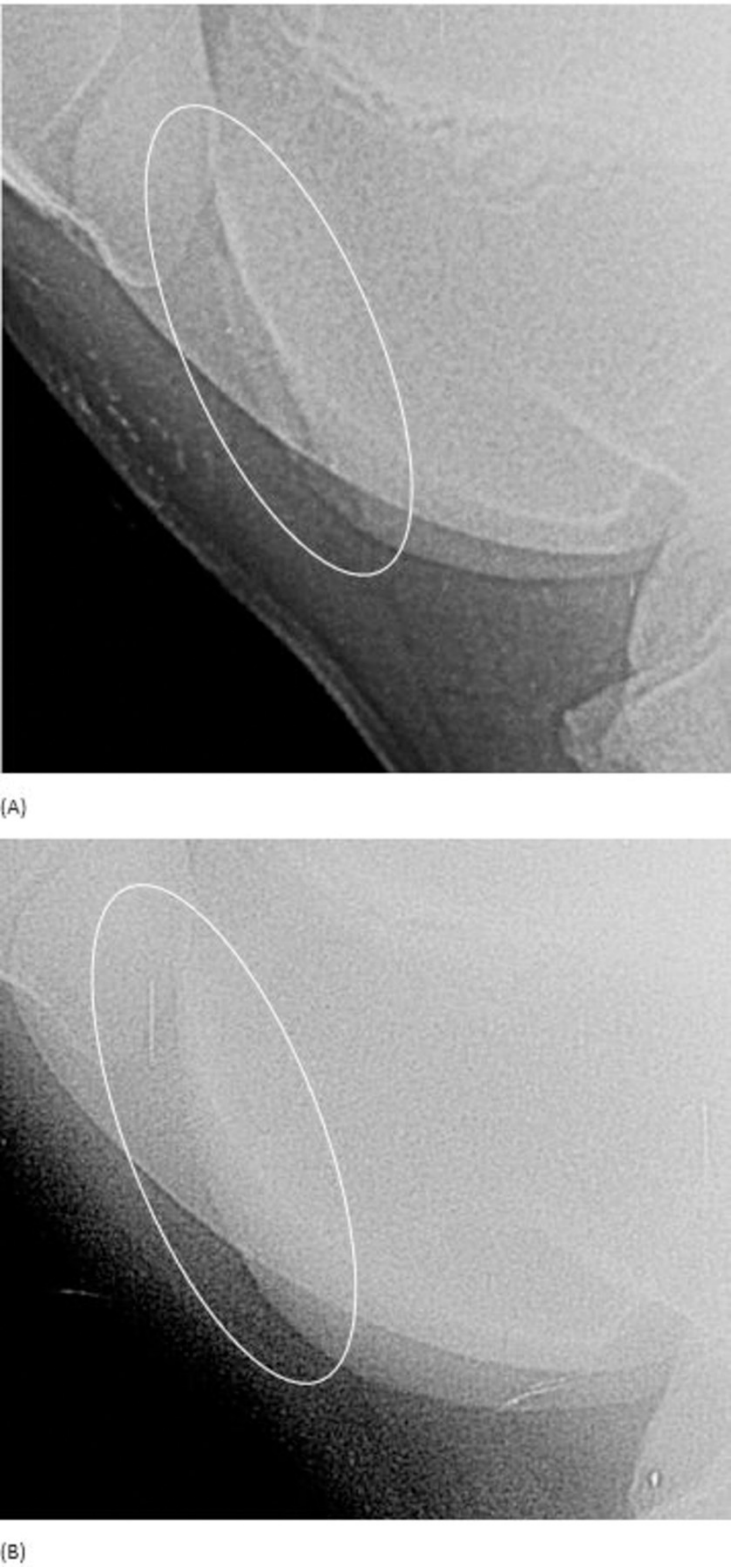
Latero-medial view of the left femoropatellar joint of a horse at 379 days (A) and 596 days (B): (A) Presence of a radiopaque zone next to a flattening of the lateral ridge of the femoral trochlea (circled) suggesting the presence of osteochondrosis dissecans. (B) The homogeneity and contour of the lateral ridge improved and was more regular. Fragments are no longer visible. The lesion was graded as osteochondrosis and considered to have improved.

2 OCD lesions were observed on first examination in the LTF and one on the second, indeed, one was considered has OC on second examination (Fig 4).

## Discussion

To our knowledge, this is the first study which describes changes in the radiographic aspect of OC(D) lesions and the appearance of OC(D) lesions in different joints in sport horses aged between 12 and 36 months.

Indeed, the evolution of developmental orthopedic pathologies between 6 and 18 months of age is well described in foals by studies in relatively large populations) [24, 32, 42]. However, there was no follow-up between both examinations. Therefore, these publications do not make it possible to establish the precise moment when lesions stop evolving. The precise dynamic aspect of OC(D) lesions was evaluated by few studies on relatively small populations of horses and dynamic characterization is scarce. Indeed, few studies have analyzed modifications of OC(D) lesions during the first and second years of life on different breeds (Trotters, Warmblood, Lusitano) [34–36]. Among these, results were not similar regarding the age of “no return” and the absence of progression/apparition of lesions in the different joints, which varied from 6 months to 12 months (except for DP P1 lesions which were not considered as OC(D) lesions). Methodologies were not comparable as the studied joints differed, the views (incidence and number) were not the same, the time frame between examinations varied and the number of horses and mainly the number of lesions were very low. However, in an article published by Lewczuk and collaborators in 2017 about osteochondrosis associated Single nucleotide Polymorphism in Polish Warmblood horses before and after a training period (average age of 1305 days), they found that a small proportion of them presented OCD lesions only after training (29% and 31% of the males were affected respectively before and after the training session and 23% versus 27% for the female group) [43]. This fact was not deeply studied, horses were classified in the group which was free of osteochondrosis if no fragment was present even in the case of shape irregularity, flattening or other disturbance was present, which can cause confusion on the presence or not of osteochondrosis within a joint. Another study, conducted on Thoroughbred horses, assessed stifle radiographic appearance through 3 examinations. This study described a small proportion of stifle radiographic appearances which evolved between the two last exams carried out at around 347 and 534 days of age [33]. However, some individuals were younger than one year old (147 to 489 days) during the examination and this study does not demonstrate the presence of evolution after one year of age.

In the study presented here, the number of individuals which recovered or developed OC(D), through radiographic diagnosis, after 12 months was statistically different from 1%. However, at the level of the joints, only the healing rate was different from 1%. Indeed, very few developed lesions after one year of age. It was considered appropriate to compare the value obtained with 1% given the financial cost of therapeutic arthroscopy, the risk of lameness associated with certain lesions and the direct impact on horses prices [44–46]. If there was a risk greater than 1% of healing or developing a lesion, it was therefore considered significant enough to be taken into account.

The prevalence and distribution of OC(D) lesions differs according to the breed and individual characteristics. It appears that heavier and taller horse breeds and individuals tend to develop more OC(D) [27, 30, 46–50]. Indeed, growth plates close earlier in light breeds and in more distal bones [34, 51–53]. Also, the thickness of the epiphyseal growth cartilage is less important and the number of vessels are lower in ponies than in horses and differ according to the joints [14–16, 54]. All of these elements therefore suggest that the evolution window of OC(D) lesions must take into account the breed of the animal and the joint evaluated.

The origin of OC(D) lesions at different joint sites remain partially understood. Indeed, in femoral trochlea lesions and 3 typical sites of tarsocrural OC(D) (medial malleolus, DIRT, lateral trochlear ridge of the talus), it is commonly accepted that they are the consequence of enchondral ossification impairment [1, 11, 54, 55]. However, the various lesions that can occur within fetlock joints still are subject of disagreement as to their origin. It is accepted that IR of the condyles of MCIII and MTIII fragments have as their origin a process of osteochondrosis [1, 16, 56]. However, in Thoroughbreds, DP P1 fragments are considered as traumatic fracture [57–59]. On the other hand, in sport horses, no sign of fracture (fracture line, peripheral bone reaction, transient or permanent joint inflammation) could be demonstrated in the context of various studies. A purely traumatic origin is therefore unlikely and an enchondral ossification impairment coupled with repeated contacts with the dorsal aspect of the MCIII/MTIII leading to fragmentation seems to be the most promising hypothesis [1, 6, 16]. Within this study, POFs have been described but not included in the prevalence. Indeed, some authors consider them as a separate entity [26, 27]. Various morphological and histological studies have, in fact, not demonstrated the presence of modifications typical of OC(D) lesions. It seems that these fragments are, in large part, related to an avulsion fracture process at the ligament insertion and/or a non-union of palmar/plantar eminence of the first phalanx [60, 61]. Nevertheless, the presence of areas of chondronecrosis at the insertion level and the fact that there seems to be a degree of heritability of POFs as well as a genetic correlation with hock OC(D) do not make it possible to establish with reliability the origin of these fragments [16, 62, 63]. In our study, one POF appeared closer to P1 on second examination, which might suggest that the lesion had regressed. It was nevertheless considered stable due to a lack of projections. In order to assess the evolution and prevalence of this type of fragments with precision, it would have been necessary to perform dorsoproximal lateral-palmaro/plantarodistal medial oblique (D35Pr45L-P/PlDiMO) and dorsoproximal medial-palmaro/plantarodistal lateral oblique (D35Pr45M-P/PIDiLO) views [34].

Previous studies have reported that the evolution of lesions between 6 and 18 months differed between joints. Indeed, in these studies, stifle lesions had a strong tendency to regress, those of the hock showed little active dynamics (the majority of lesions remaining similar) and finally, in fetlocks, the process seemed quite dynamic with a higher number of lesions at 18 months than at 6 months [24, 42]. Compared to the observations carried out in our cohort, the tarsocrural joint seemed the most stable. The femoropatellar joint evolution leads towards a regression/disappearance of lesions (2 evolved as a regression or disappearance). Concerning the evolution of fetlock lesions, 1 out of 5 were favorable and the other 4 were unfavorable. However, the relatively low number of OC(D) per joint in the present study does not allow for reliable conclusions as to the type of evolution specific to each joint. It would be relevant to consider this analysis on a higher number of lesions.

The agreement between observers in detecting and grading OC(D) and evolutionary status varied from fair to almost perfect (κ = 0.40 to 0.84). These results seem quite similar to the results of previous studies (37–39). Most disagreements when grading lesions between Vet 1 and Vet 2 appeared in subtle lesions—usually, Vet 1 considered the joint to be healthy whereas Vet 2 considered that there was an OC lesion (10 out of 12 disagreements). Disagreements between Vet 2 and Vet 3 followed the same pattern: among the 12 disagreements, Vet 2 detected 11 OC lesions while Vet 3 considered the joints healthy. Regarding the evolutionary status disagreements between Vet 1 and Vet 2, all of them (2 disagreements) concerned joints in which Vet 1 detected no evolution and Vet 2 saw an improvement. Vet 3 judged the evolution to be less significant or absent in 1 out of 2 disagreements with Vet 2.

Our study also aims at achieving therapeutic optimization of OC(D) lesions. Various sources recommend operating affected individuals at one year old [5, 64, 65]. Indeed, sports prognosis seems to be correlated with size, location, type of lesions, presence of secondary lesions (which are frequent despite an absence of clinical signs) and operation age [6, 57, 65]. However, in our study, the evolution of some lesions suggests that, in the absence of clinical signs, surgery should not be performed as early as 12 months because of the possible spontaneous improvement of lesions.

### Limitations of the study

The study presented here contains a certain number of limitations. First, the population studied was relatively limited and therefore so was the number of lesions. This limitation implies that conclusions should be considered with caution. We can speculate that the absence of a significant effect of different parameters, such as age, at examinations as well as the duration between them is inherent to the small number of horses and that a study on a larger population would highlight the presence of significant effects. A second main limitation is that, in the context of the evolution of OC(D) lesions, although the protocol used here is considered indicated for OC(D) screening for these joints [30], there is a certain range of radiographic images for which it is not possible to discriminate with certitude the presence or absence of OC(D) lesions [66]. This is why, in the context of accurate diagnosis and/or suspicion of OC(D) lesions, the different joints are subjected to several projections in order to increase sensitivity and diagnostic specificity [34, 67, 68]. Moreover, as discussed above in context of POF, some lesions cannot be detected or well analyzed though this protocol, such as medial malleolus and lateral malleolus in tarsocrural joint, medial trochlear ridge condyle cysts of the femur and DP P1 OC(D) [38, 66]. However, the number of views taken per foal was limited here in order to respect the “ALARA” (As Low As Reasonably Achievable) radiation protection concept and the security of each operator. Moreover, x-raying the limbs of these foals—most often little handled—even if they are sedated, is not without risk. In order to accurately assess the prevalence of lesions as well as their evolution, further studies based on multiple views of each joint should be conducted. A third limitation resides within the frame of time between examinations. The minimum time between examinations was 4.5 months (142 days) and the maximum 13.5 months (405 days), which is more than double the time. Futures studies with standardized time between examinations are required to assess accurately the dynamic of osteochondrosis lesions. The influence of metabolism and management of foals was highlighted in previous studies—both in terms of feeding and foal management—on the appearance, progression, regression and disappearance of OC(D) lesions [24, 33, 69, 70]. In the context of our retrospective study, the type of horse management was probably representative of sport horse breeding habits in Wallonia, given the diversity of breeding places from which the animals came (as described in previous study [24]). As for future perspectives, it would therefore be interesting to carry out research integrating the management aspect of individuals in the dynamic of OC(D) after 12 months old, depending on the joint concerned. Another limitation concerns the gradation of radiographs. When analyzing radiographs, lesions were graded in two of the studies cited above on a scale from 1 to 4. We did not use this grading system for several reasons: the use of different systems (2 Computed Radiographs and 1 Direct Radiograph) in order to perform and process radiographs, incidences which were not always exactly similar and the variability of inter-observer agreement in gradation of different osteo-articular pathologies in horses [67, 71]. Therefore, the hypothesis of our study being to demonstrate the presence of an evolution of OC(D) lesions within different joints in Warmblood horses after the age of 12 month—which did not seem to happen according to previous publications—and in order not to over-diagnose their presence, considering the evolution of lesions according to a grading system such as appearance/disappearance/deterioration/improvement and in case of doubt (disagreement between observers) considering them as stable, was deemed most appropriate.

## Conclusions

Based on this study, although it is not possible to report a detailed time frame of accurate dynamics, we can report in detail that is obvious under the age of 365 days for metacarpo and metatarsophalangeal, tarsocrural and femoropatellar joints. Therefore it seems that in the absence of clinical signs, surgery on OC(D) lesions in horses less than one year old is not necessary and indicated because of possible spontaneous resolution of those lesions. Further studies are necessary to characterize the dynamics and influencing factors of osteochondral lesions.
